# Adult offspring of high-fat diet-fed dams can have normal glucose tolerance and body composition

**DOI:** 10.1017/S2040174414000154

**Published:** 2014-03-10

**Authors:** K. M. Platt, R. J. Charnigo, K. J. Pearson

**Affiliations:** 1 Graduate Center for Nutritional Sciences, College of Medicine, University of Kentucky, Lexington, KY, USA; 2 Department of Biostatistics, College of Public Health, University of Kentucky, Lexington, KY, USA

**Keywords:** mice, obesity, pregnancy, programming

## Abstract

Maternal high-fat diet consumption and obesity have been shown to program long-term obesity and lead to impaired glucose tolerance in offspring. Many rodent studies, however, use non-purified, cereal-based diets as the control for purified high-fat diets. In this study, primiparous ICR mice were fed purified control diet (10–11 kcal% from fat of lard or butter origin) and lard (45 or 60 kcal% fat) or butter (32 or 60 kcal% fat)-based high-fat diets for 4 weeks before mating, throughout pregnancy, and for 2 weeks of nursing. Before mating, female mice fed the 32 and 60% butter-based high-fat diets exhibited impaired glucose tolerance but those females fed the lard-based diets showed normal glucose disposal following a glucose challenge. High-fat diet consumption by female mice of all groups decreased lean to fat mass ratios during the 4th week of diet treatment compared with those mice consuming the 10–11% fat diets. All females were bred to male mice and pregnancy and offspring outcomes were monitored. The body weight of pups born to 45% lard-fed dams was significantly increased before weaning, but only female offspring born to 32% butter-fed dams exhibited long-term body weight increases. Offspring glucose tolerance and body composition were measured for at least 1 year. Minimal, if any, differences were observed in the offspring parameters. These results suggest that many variables should be considered when designing future high-fat diet feeding and maternal obesity studies in mice.

## Introduction

Obesity is rampant in the United States with potential for annual economic ramifications that could surpass $209.7 billion.^1^ ^,^ ^2^ No intervention has been identified that can effectively decrease obesity rates in a large proportion of the population, and an overweight/obesity prevalence approaching 90% has been predicted should trends continue.^3^ Among other complications, obesity increases the risk for developing type 2 diabetes.^4^ ^,^ ^5^ Since up to half of women of childbearing age in the United States are overweight or obese,^6^ countless future generations could suffer deleterious consequences because of *in utero* exposure to maternal obesity and diabetes.

Developmental programming, recognized for over 20 years, is the concept that the uterine environment to which a developing fetus is exposed can impact its response to stimuli throughout the duration of its postnatal life.^7^ ^,^ ^8^ Retrospective studies have indicated that babies exposed to under-nutrition during gestation have increased incidence of diabetes, cardiovascular disease and certain cancers later in life.^9^ Conversely, *in utero* exposure to excess nutrients leaves offspring more susceptible to obesity, diabetes and other complications.^10^ ^,^ ^11^ One can easily visualize the cycle of obesity that can be created.

Animal studies, particularly in rodents, are widely used to explore the offspring consequences of maternal high-fat diet consumption, a common model for over-nutrition. Studies report a plethora of detrimental findings in mouse offspring as a consequence of maternal high-fat feeding, including increased risk of fatty liver disease, bone malformation, glucose intolerance and insulin resistance.^12^ ^–^ ^15^ Maternal obesity and high-fat diet consumption and their influence on offspring obesity outcomes are less clear. Some studies suggest an increase in rat offspring obesity,^16^ ^–^ ^18^ while another indicates no effect.^19^ Experiments in mice, in general, suggest an increase in offspring weight due to maternal high-fat diet intake and/or obesity.^20^ ^–^ ^22^ Several studies indicate that impaired glucose tolerance can be induced in offspring as a consequence of maternal high-fat diet feeding in both rats^23^ ^–^ ^25^ and mice.^21^ ^,^ ^26^

Many studies compare non-purified, cereal-based-diet (commonly referred to as laboratory chow)-fed control animals to purified high-fat diet-fed animals.^27^ ^–^ ^35^ Cereal-based diets are those that are primarily sourced from natural ingredients such as grains and meals.^36^ ^–^ ^38^ Cereal-based diets often have variable composition, therefore introducing nutrients and phytochemicals not represented in a purified high-fat diet.^39^ There is even notable variability between different batches of the same formula cereal-based diet.^40^ Conversely, many purified diets, including the ones used in the current study, substitute sucrose in place of fat when formulating the control diet. High sucrose diets fed perinatally in mice may introduce offspring complications of their own.^41^ Therefore, a perfect solution may be difficult to find.

For this study, we set out to find a readily available commercial high-fat diet that could be fed to female ICR mice before and during pregnancy and nursing that would induce impaired glucose tolerance and/or altered body composition in offspring when compared with purified control diet-fed animals. We chose two lard-based high-fat diets (45 and 60% by calories) and two butter-based high-fat diets (32 and 60% by calories) from Research Diets Inc. (New Brunswick, NJ, USA). Control animals received either the lard- or butter-based diet containing 10–11% fat calories. We hypothesized that male and female offspring born to high-fat diet-fed dams would have impaired glucose tolerance and show increased body weight and obesity during adulthood. Male and female offspring from all dietary groups were followed for at least 1 year with minimal effects on glucose tolerance, body weight and body composition.

## Materials and methods

The current study was carried out according to a protocol approved by the University of Kentucky Institutional Animal Care and Use Committee. Fourteen-week-old female ICR mice (*n*=120) were purchased from the vendor (Taconic Farms, Germantown, NY, USA) after they had delivered one litter at their facility. Animals were acclimated to controlled temperature (21–24°C), humidity and light/dark cycle (14/10 h) for 1 week after arrival to the University of Kentucky and maintained under these conditions for the duration of the study. All cages contained standard chipped wood substrate (Sani-Chip, Harlan-Teklad, Madison, WI, USA), a cotton Nestlet^TM^ for bedding (Ancare, Bellmore, NY, USA) and a plastic mouse igloo (Bio-Serv, Frenchtown, NJ, USA). After acclimatization, the females were housed four per cage and separated into six groups approximately balanced by body weight (*n*=20 per group). Animals received one of six commercially available purified diets (Research Diets Inc.). The three lard-based diets were D12450B (10% fat calories, 3.9 kcal/g), D12451 (45% fat calories, 4.7 kcal/g) and D12492 (60% fat calories, 5.2 kcal/g). The three butter-based diets were D12489B (11% fat calories, 3.9 kcal/g), D12266B (32% fat calories, 4.4 kcal/g) and D02101801 (60% fat calories, 5.3 kcal/g). See Supplementary Tables S1 and S2 for detailed dietary information. There are some key differences in the diets. For instance, while the lard diet used consistent soybean oil contents for all three diets, the butter diets used widely variable amounts of corn oil instead. They also contained two different mineral mixes. Dam body weight and food consumption were recorded weekly for the duration of the study. Food consumption data were collected per cage and averaged based on the number of mice in that cage to obtain a daily intake value. During the 4th week of diet treatment (days 23–25 of diet), animals were subjected to glucose tolerance testing and body composition analysis (EchoMRI^TM^; Echo Systems Inc., Houston, TX, USA). After the 4th week of diet treatment, animals were bred to ICR males. For breeding purposes, two females were introduced into a cage containing one male to cohabitate continuously for 6 days and nights before removal of the male (thus the male also consumed the respective diets of the female mice during mating). One week after removal of the male, females were split into individual cages and remained single housed for the duration of pregnancy and suckling. Offspring were counted (Table 1) and culled to eight pups per litter on postnatal day 2. Alternatively, on day 2, pups in litters of less than eight were cross-fostered after marking for identification in order to balance litters. Offspring weights were recorded on postnatal days 7, 14 and 21. All dams were switched to the control diet within the fat source group of their current diet (60% lard were switched to 10% lard; 32% butter were switched to 11% butter, etc.) at postnatal day 14. This was in an attempt to minimize the opportunity for the offspring to consume the high-fat diet themselves. Offspring were implanted with microchip transponders (BioMedic Data Systems Inc., Seaford, DE, USA) for identification between days 17 and 19 and weaned onto the 10–11% control diet consumed by their dam on postnatal day 21. Offspring body weight was recorded biweekly for more than 1 year. Body weight data represents one randomly chosen offspring per sex per treatment group.

**Table 1:** Number of litters born, average pups per litter and litters weaned out of those bred

Group	Females bred	Litters born	Pups/litter (mean±s.e.m.)^a^	Litters weaned
10% lard	20	18	10.9±0.6	17
45% lard	20	15	10.6±0.6	15
60% lard	20	14	8.6±0.9	11
11% butter	20	17	10.1±0.4	16
32% butter	20	12	9.4±0.6	10
60% butter	20	12	9.8±0.4	12

a One-way analysis of variance revealed a significant dose effect regarding pups per litter within the lard-fed animals, but this difference was not significant upon *post-hoc* analysis.

### Glucose tolerance testing

During the 4th week of diet treatment, female mice were fasted for 3 h and blood glucose was recorded via tail nick using a standard glucose testing meter (Bayer Breeze2; Bayer HealthCare LLC, Tarrytown, NY, USA). Animals received a bolus of 2 g dextrose (Bimeda^®^, Oakbrook Terrace, IL, USA) per kg body weight via oral gavage. Blood glucose readings were repeated from the same tail nick 15, 30, 60 and 120 min after glucose was administered. At 6 weeks and 3 months of age, offspring from all groups were fasted for 3 h and subjected to the same glucose tolerance testing protocol, except dextrose was delivered by intraperitoneal injection. At 8, 10 and 12 months of age, offspring were subjected to glucose tolerance testing with dextrose administered via oral gavage, but otherwise following the same procedure as described. At 15 months of age, offspring were fasted overnight (18 h) and underwent the intraperitoneal glucose tolerance testing procedure but were injected with 2.67 g dextrose/kg body weight. Varied fasting times, doses and alternate routes of delivery were used in order to attempt to elucidate any potential differences in glucose tolerance. Offspring glucose tolerance represents no more than one pup per litter per sex at each time point, though not necessarily all the same animals at every time point.

### Body composition analysis

Dams were subjected to EchoMRI during the 4th week of dietary treatment. Briefly, animals were gently restrained in a plastic tube designed specifically for the EchoMRI. The tube was inserted into the EchoMRI and the animal was analyzed for fat and lean mass as well as free and total water content. A portion of the animal’s mass is unaccounted for (hair, bone and connective tissue). The analysis for one mouse is complete in ~2 min. Offspring body composition was analyzed at 7 weeks, 3, 10 and 12 months of age following the same procedure. Offspring body composition represents no more than one pup per litter per sex at each time point, though not necessarily all the same animals at every time point.

### Statistics

**Fig. 1:**
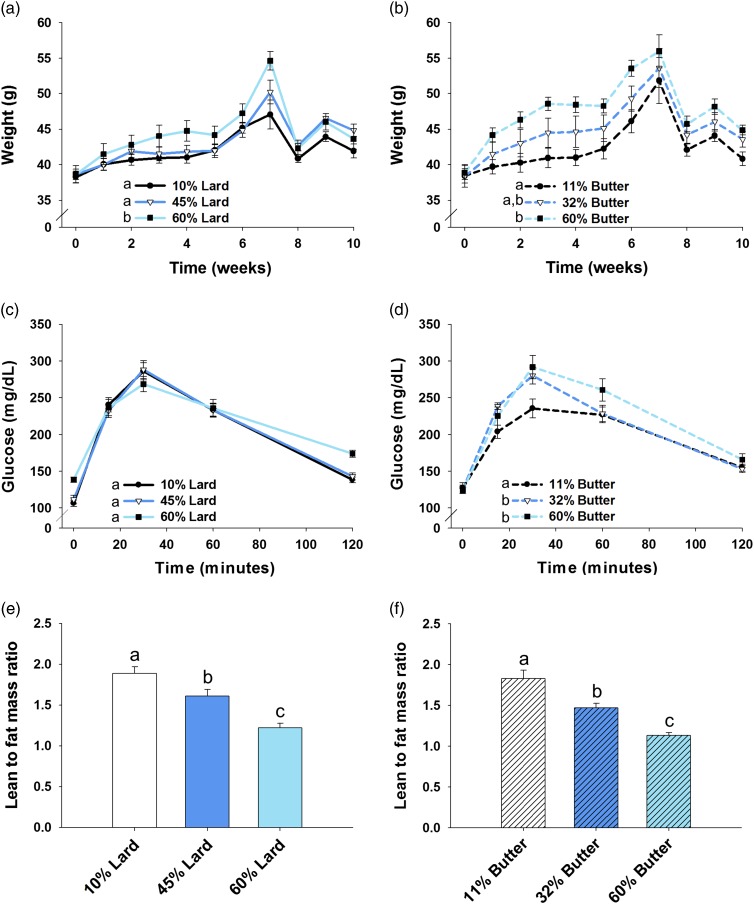
Dam body weight, glucose tolerance and body composition. Body weight (*a*, *b*) for dams that weaned litters is shown. Weight was increased in both 60% lard and 60% butter high-fat diet-fed animals compared with their respective control group (*n*=11–17 for lard, *n*=10–16 for butter). Glucose tolerance was not significantly impaired in lard animals (*c*) the week before mating, but glucose disposal was impaired in both 32 and 60% butter animals (*d*) (*n*=16 per group). Lean to fat mass ratio was decreased in an incremental fashion before mating for both 45 and 60% lard (*e*) and 32 and 60% butter females (*f*) (*n*=20 per group). Groups not sharing a common letter (‘a’, ‘b’ or ‘c’) in the legend of the graph or on the graph itself are significantly different. Error bars indicate s.e.m.

**Fig. 2:**
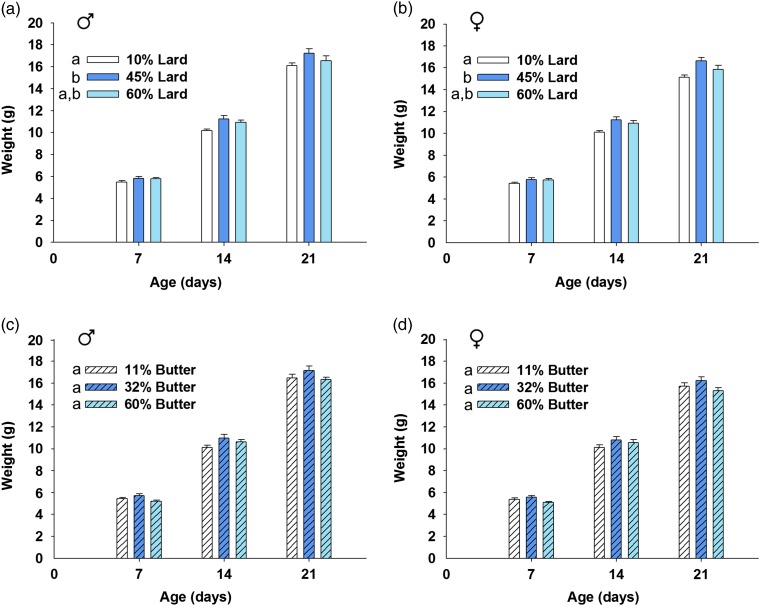
Offspring weight before weaning. Pups were weighed 7, 14 and 21 days after birth, and the average pup weight by sex was calculated for each litter. Weights were increased for male and female offspring born to 45% lard-fed dams before weaning compared with controls (*a*, *b*). Weight was not significantly changed for male or female butter offspring before weaning (*c*, *d*). *n*=10–17 lard litters per group and *n*=10–16 butter litters per group. Groups not sharing a common letter (‘a’ or ‘b’) in the legend of the graph are significantly different with respect to the area under the curve of the trajectory from 7 to 21 days. Error bars indicate s.e.m.

**Fig. 3:**
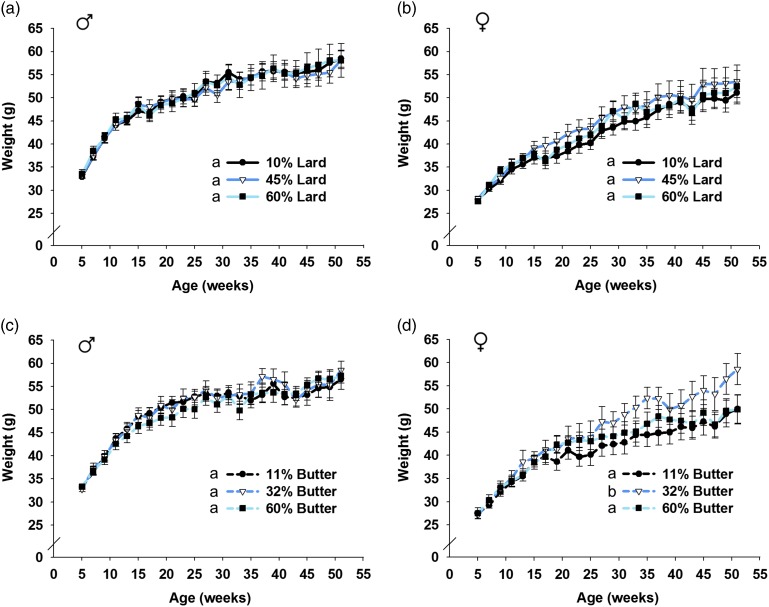
Offspring weight after weaning. Male and female offspring were weighed biweekly and results from the first year are shown. There was no significant effect on lard offspring weight as a consequence of maternal diet (*a*, *b*). Male butter offspring weight was not significantly affected (*c*), but maternal 32% butter diet increased female offspring weight (*d*). *n*=9–15 for male lard (*a*), 11–17 for female lard (*b*), 10–16 for male butter (*c*) and 9–14 for female butter (*d*). Groups not sharing a common letter (‘a’ or ‘b’) in the legend of the graph are significantly different. Error bars indicate s.e.m.

**Fig. 4:**
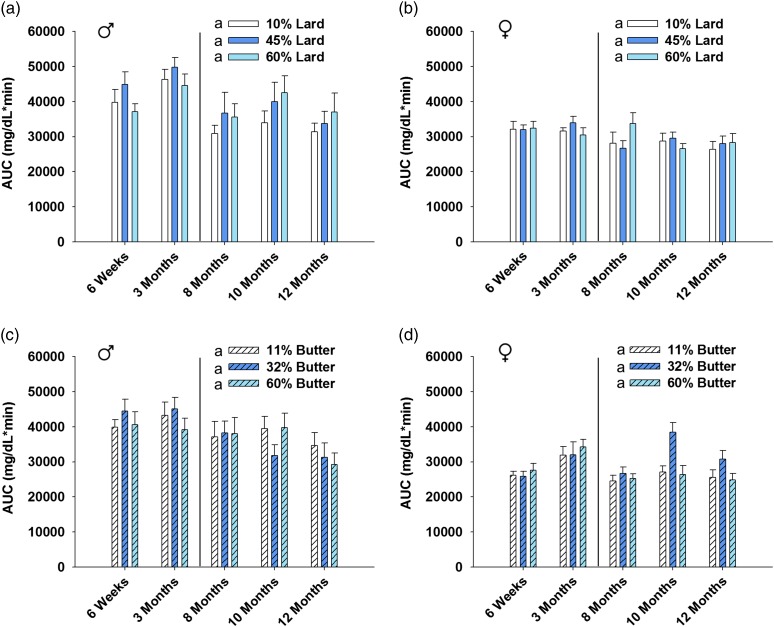
Offspring glucose tolerance area under the curve (AUC). Glucose tolerance in the mice was measured at multiple time points and is summarized in the graphs by showing AUC at each age. Values to the left of the vertical line (6 weeks, 3 months) were obtained after an intraperitoneal glucose tolerance test, while those on the right of the line (8, 10 and 12 months) were obtained from an oral glucose tolerance test at 2 g/kg body weight after a 3 h fast. Neither maternal lard (*a*, *b*) nor butter (*c*, *d*) high-fat diet significantly altered offspring glucose tolerance AUC over time. *n*=6–10 per sex per group at each time point. The AUC at each age was calculated using all of the data points acquired over the 2 h time course of the glucose tolerance test administered at that age. Error bars indicate s.e.m.

**Fig. 5:**
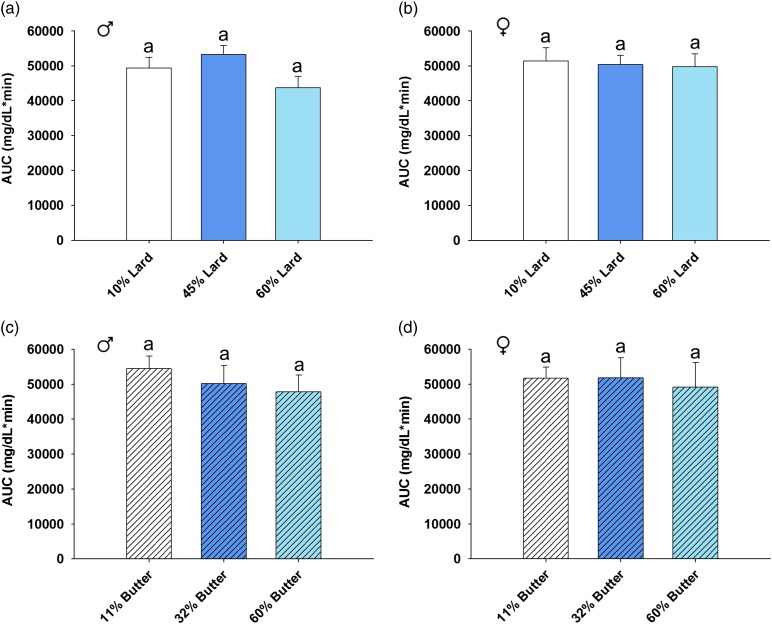
Glucose tolerance for aged offspring after an overnight fast. Offspring were subjected to an intraperitoneal glucose tolerance test at 15 months of age. Animals received a glucose dose at 2.67 g/kg body weight after an 18 h overnight fast. Area under the curve (AUC) was calculated to summarize the 2 h time course for the blood glucose trajectory. *n*=5–10 per sex per group. Neither lard (*a*, *b*) nor butter (*c*, *d*) AUC was significantly altered by maternal high-fat diet. Error bars indicate s.e.m.

**Fig. 6:**
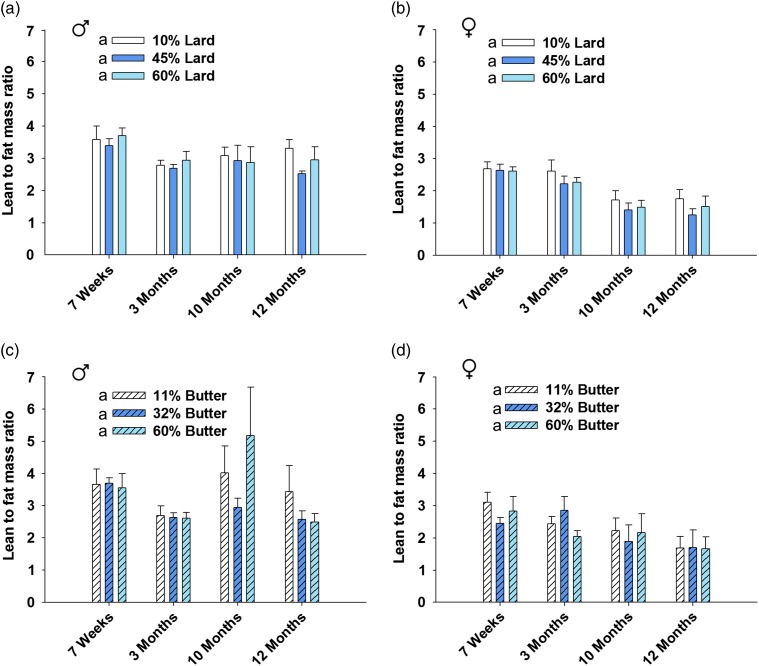
Offspring lean to fat mass ratio. EchoMRI body composition analysis was completed at 7 weeks, 3, 10 and 12 months of age. There was no significant impact on lard (*a*, *b*) or butter (*c*, *d*) offspring lean to fat mass ratio for the duration of the study. *n*=7–17 per sex per group at each time point. Error bars indicate s.e.m.

Data analysis was completed using version 9.3 of SAS (SAS Institute, Cary, NC, USA) and version 11.0 of SigmaPlot (SigmaPlot Software, San Jose, CA, USA). Fisher’s exact test was employed to analyze the number of litters born and weaned out of those bred (Table 1). The number of pups per litter was analyzed using one-way analysis of variance (ANOVA) followed by Bonferroni-adjusted *post-hoc* test where applicable (Table 1). Dam lean to fat mass ratio and 15 month offspring area under the curve (AUC) was analyzed via one-way ANOVA with Bonferroni-adjusted *post-hoc* testing (Fig. 1e, 1f and 5). For outcomes assessed repeatedly over an experimental time course (Fig. 1c and 1d) or age (Fig. 1a, 1b, 2, 3 and 6), linear mixed models were fit (via PROC MIXED in SAS) to relate outcome trajectories to the different doses of lard and butter in the diets, with random effects for individual mice (or litters for pup weights) to account for possible correlations among repeated assessments on the same mice (or litters). Bonferroni-adjusted *post-hoc* tests sought to identify pairwise differences between doses with respect to the change in the trajectory from baseline (Fig. 1a, 1b and 3) or the AUC of the trajectory (Fig. 1c, 1d, 2 and 6). The glucose tolerance tests for offspring yielded doubly repeated measures data: the tests were conducted at multiple ages, and at each age there were multiple data points acquired over the 2 h time course. The data points at each age were summarized by an AUC score (via the ‘Area Below Curves’ function in SigmaPlot), and then the AUC scores at various ages were treated as outcomes in linear mixed modeling. Bonferroni-adjusted *post-hoc* tests then sought to identify pairwise differences between doses with respect to the AUC of the trajectory (in effect, an AUC of an AUC; Fig. 4). Statistical significance was defined by a *P*-value<0.05.

## Results

### Dams

There was a significant dose effect for change in dam body weight over time as analyzed by linear mixed model ANOVA for the lard- and butter-based high-fat diet groups (*P*<0.001 and *P*=0.016 for lard and butter, respectively). Dam body weight increased significantly as a consequence of consumption of either of the 60% fat diets compared with their respective 10–11% control diet (*P*<0.001 for lard; *P*<0.001 for butter by *post-hoc*) (Fig. 1a and 1b). Dams fed the 60% lard diet were also heavier than 45% lard (*P*=0.002 by *post-hoc*), though neither the 45% lard nor 32% butter were heavier than their respective controls. Body weight differences were not a consequence of differential calorie consumption as all animals consumed similar numbers of calories on average compared with their respective control diet group when analyzed by linear mixed model ANOVA for dose effect change over time (*P*=0.551 for lard; *P*=0.879 for butter) (data not shown). Neither resting energy expenditure nor activity levels were measured, but changes in these characteristics could have led to increased body weight in the 60% fat-fed female mice.

During the 4th week on the diets, glucose tolerance and body composition were measured in the female mice. The glucose tolerance test data were analyzed by linear mixed model ANOVA, and there were trends for dietary dose effects toward increased blood glucose in lard high-fat diet-fed females both at the initial time point and over time, although neither reached significance (*P*=0.060 for fasting; *P*=0.083 for change over time) (Fig. 1c). There was a significant dietary dose effect for an increase in glucose over time in the butter-based high-fat diet–fed female mice (*P*<0.001 by linear mixed model ANOVA). Following *post-hoc* analysis, it was determined that the 32 and 60% butter-based diet-fed animals had impaired glucose disposal compared with the 11% fed controls (*P*=0.011 for 32%; *P*=0.002 for 60%) (Fig. 1d). In addition, the AUC for the glucose tolerance test was analyzed by one-way ANOVA (data not shown). Lard animals AUC was not significantly affected by the diet treatment, but butter animals consuming the 60% diet had increased AUC compared with 11% controls (*P*=0.006). Both high-fat lard diets decreased lean to fat mass ratio in an incremental fashion in the female mice before mating when compared with their respective control group (*P*=0.029 for 45% and *P*<0.001 for 60% compared with 10%; *P*=0.001 for 45% compared with 60%) (Fig. 1e). The same incremental decrease was present in the butter-diet-fed animals (*P*=0.002 for 32% and *P*<0.001 for 60% compared with 11%; *P*=0.004 for 32% compared with 60%) (Fig. 1f).

Data concerning litters born, number of pups born per litter and litters weaned are presented in Table 1. There were no significant decreases in number of litters born or weaned out of those bred in the high-fat lard or butter diets compared with their respective control diets, but the 10–11% fat diets did have the highest number of litters born and weaned. There was a significant dose effect for number of pups per litter for lard-diet-fed animals (*P*=0.044), but the difference did not reach significance upon *post-hoc* analysis (*P*=0.053 for 60% lard compared with 10% lard). The number of pups born per litter in the butter cohort was not significantly affected by the fat percentage in the diet (*P*=0.565). It is possible that a larger number of breeding females would have established negative litter outcomes for the high-fat diet-fed dams compared with the controls.

### Offspring

Pre-weaning pup weights per litter were significantly affected by dose of high-fat diet over time for female and male offspring born to lard-based-diet-fed dams (*P*=0.008 and *P*<0.001 for male and female lard, respectively, by linear mixed model ANOVA). Pup weight was significantly increased before weaning for pups born to 45% lard-based-diet-fed dams compared with 10% controls (*P*=0.007 for lard male pups and *P*<0.001 for lard female pups), but those from 45% lard-fed dams did not significantly differ from pups born to 60% lard-fed dams (Fig. 2a and 2b). Neither male nor female pups born to 60% lard-fed dams weighed significantly more than 10% fat-fed control pups, but there was a strong trend toward increased weight in the female offspring born to 60% lard-fed dams compared with female offspring born to control diet-fed dams (*P*=0.050). Male and female offspring born to butter-based-diet-fed dams had no change in mean pup weight per litter before weaning compared with the 11% controls (*P*=0.109 and 0.263 for butter-based males and females, respectively) (Fig. 2c and 2d). Body weight over the 1st year is shown in Fig. 3. No differences were observed in male or female lard offspring by linear mixed model ANOVA (*P*=0.821 for males; *P*=0.260 for females) (Fig. 3a and 3b). Similarly, male butter offspring weights were not significantly different (*P*=0.163) (Fig. 3c). Female butter offspring born to dams that consumed 32% fat diet had increased body weight as they aged compared with offspring from 11 or 60% dams (*P*<0.001 for both comparisons) (Fig. 3d) even though the body weights for this group did not differ significantly pre-weaning. The cause of this increased body weight in the adult female offspring is unknown but was not due to increased food consumption as average daily food intake was not significantly different in those females born to the 32% fat-fed dams compared with control dams (data not shown). It could have resulted from a decrease in metabolic rate and/or activity in the offspring born to the 32% fat-fed dams, but these parameters were not measured.

AUC was calculated for each of the glucose tolerance tests up to 1 year of age, and the overall differences were assessed. There were no significant differences in AUC for lard males (*P*=0.138), lard females (*P*=0.876), butter males (*P*=1.00) or butter females (*P*=0.159) compared with the respective control diet groups (Fig. 4). In order to determine whether a short fasting time could have masked any differences, a glucose tolerance test was completed with a higher dose of glucose after an overnight fast in the offspring when they were 15 months of age (Fig. 5). AUC was not significantly different in any sex or dietary group at this advanced age as determined by one-way ANOVA.

Similarly, lean to fat mass ratio was analyzed across all time points, and no significant effects were observed for lard males (*P*=0.904), lard females (*P*=0.457), butter males (*P*=0.249) or butter females (*P*=0.833) (Fig. 6). Of note, the offspring glucose tolerance tests and body composition data represent no more than one pup per litter per sex at each time point. Though, not all of the same animals were included at each time point in Figs 4 and 6. Thus, these data do not represent trajectories of progressions for individual animals but rather estimates of mean-level trajectories of progression within populations of animals.

## Discussion

The current study found minimal effects in offspring body weight, glucose handling and body composition as a consequence of maternal consumption of a variety of commercially available high-fat diets. Although some differences in lard pup weight before weaning were apparent, these differences disappeared after weaning. After weaning, the female offspring born to 32% butter dams were the only animals to have a significantly increased weight, but this increased weight did not correspond to any significant change in glucose tolerance or body composition. We do not have an explanation for the increased weight in this group as we did not monitor energy expenditure or activity levels. While the lack of detrimental effects was surprising, we propose a number of explanations. We will discuss several of these possibilities, including the use of purified diet for the control groups, the use of the ICR stock of mice, the use of primiparous females and the removal of the high-fat diet at postnatal day 14.

Irrespective of rodent species or outcome of interest, it is common practice to use cereal-based diets as the control diet for high-fat maternal feeding studies.^27^ ^–^ ^35^ Cereal-based diets have some characteristics that researchers should consider when using them. For example, they contain phytoestrogens from the plant material used in their production. Phytoestrogens have been shown to impact many body systems, including cardiovascular, reproductive, skeletal and immune systems.^42^ ^–^ ^45^ They have been studied in the context of various disease states, including obesity.^46^ The consequence of maternal/perinatal isoflavone exposure has been an area of active study.^47^ ^–^ ^50^ Articles have been published warning about the potential impact that phytoestrogens may have on animal health in research studies.^39^ While diet manufacturers minimize the phytoestrogen content of their cereal-based diets when possible, the only way to entirely eliminate them is to use a purified diet. Future studies should directly compare cereal-based diet-fed animals to fat matched purified diet-fed animals to explore the impact, if any, on glucose tolerance and body composition. It is entirely possible that many of the notable offspring effects in the literature are due to cereal-based *v*. purified diet comparisons rather than dietary fat or lean *v*. obese dam comparisons.

In the current study, we chose to use purified, commercially available control diets as the controls for the high-fat diets. One notable shortcoming of the control diet used in the current study is that the fat difference was made up by adjusting the sucrose concentration (among other adjustments) in the diet. High sucrose diets have been shown to cause an increase in pup weight in pre-weaning rats and mice,^41^ ^,^ ^51^ as well as impairments in female mouse offspring glucose tolerance.^41^ The 10% lard-based diet contains around 35% sucrose by calories, while the 45 and 60% fat diets contain around 17 and 7% sucrose, respectively. The 11 and 32% butter-based diets both have ~25% sucrose, where the 60% butter diet offers only 9% sucrose. It is interesting that the two groups with comparable sucrose content (the 11 and 32% butter animals) are the groups that exhibited significant differences in female offspring body weight. Therefore, the variation of sucrose in the diets used in the current study could have masked potential offspring differences. The manufacturer currently offers formulations for the 10% fat diet (product numbers D12450H and D12450J) that are sucrose matched to the 45 and 60% lard diets, and they also offer a control diet that eliminates sucrose and features corn starch instead (D12450K). Future studies should use sucrose-matched diets or custom designed, content-matched diets instead of commercially available ones.

The ICR (CD1) mouse is a large, outbred stock of albino animals. We have previously described some of the breeding differences between ICR and C57BL/6 mice.^52^ The ICR animals are better breeders that deliver large litters (~10–11 pups per litter) and provide excellent maternal care. These are among the reasons that we use this strain frequently, and the large litter size allows for culling litters to control for postnatal food availability. However, some differences have been documented between the ICR stock compared with other strains regarding glucose tolerance, lung function and drug response.^53^ ^–^ ^55^ Future studies should consider the impact that mouse strain/stock may have on various outcomes of interest.

The current study used ICR females that had previously delivered one litter at the vendor before delivery to the University of Kentucky. This choice was made because we would anticipate that experienced breeding females would viably reproduce. Further, one would expect that dams delivering their second litter would provide superior care and nutrition to their pups.^56^ However, studies have shown deleterious effects of multiple pregnancies, both on the dam and on the offspring born to that dam.^57^ It is therefore possible that the controls and high-fat diet-fed female mice were already heavier than virgin females would have been, thus limiting detectable effects in the offspring. Future studies should be aware of the parity of the animals in use. While this is commonplace in developmental programming studies, other fields do not always account for the number of times a mouse has birthed litters.

It is known that maternal high-fat diets can have differential effects on offspring depending on the timing of exposure.^35^ Many high-fat diet and developmental programming studies leave the dams on the experimental diet throughout nursing until the pups are weaned.^20^ ^–^ ^22^ ^,^ ^26^ In our study, we chose to remove the high-fat diet from the dams when the pups reached 14 days of age. Our intent was to minimize the offspring opportunity to consume the high-fat diet directly, as mouse pups begin nibbling solid foods before weaning. Future studies should investigate outcomes based on the time at which high-fat diet feeding to the dams is terminated during the early postnatal period.

In conclusion, the lack of effects that we have observed in the offspring in the current study is important. While butter high-fat diet-fed dams had impaired glucose tolerance, and all high-fat diet-fed dams exhibited decreased lean to fat mass ratio when compared with their respective control group, the male and female offspring in the current study had no significant impairments in long-term glucose tolerance and no significant differences in body composition. Future studies should take into account the variables discussed herein that may have contributed to the results of the current study. Additional considerations, including the age of the breeders and the duration of dietary treatment before mating, could also play a role in the lack of offspring effects. Female mice in our study were fed the high-fat diets for a relatively short period before mating (4 weeks) and then throughout pregnancy and the majority of nursing. A longer pre-feeding before mating could possibly contribute to detrimental effects in the offspring that we did not observe as part of our study. In addition, the same maternal study design could be used with more sensitive measures of glucose handling in the offspring (such as hyperinsulinemic euglycemic clamp). This study highlights important questions that should be posed during the design of future developmental programming studies in mice and other models, and researchers should be cognizant of these potential issues as we move forward.

## Supplementary Material

Supplementary material To view supplementary material for this article, please visit http://dx.doi.org/10.1017/S2040174414000154
