# Supplementary material for: Adult offspring of high-fat diet-fed dams can have normal glucose tolerance and body composition
Source: J Dev Orig Health Dis. 2014 Mar 10;5(3):229–39. doi: 10.1017/S2040174414000154 (PMC4098028; doi:10.1017/S2040174414000154)
Supplement: Supplementary material — To view supplementary material for this article, please visit http://dx.doi.org/10.1017/S2040174414000154 [file S2040174414000154sup001.doc]

**Supplementary Table S1 │ Lard Diet Composition**

|  | **10%** | | **45%** | | **60%** | |
| --- | --- | --- | --- | --- | --- | --- |
|  | g% | kcal% | g% | kcal% | g% | kcal% |
| Protein | 19 | 20 | 24 | 20 | 26 | 20 |
| Carbohydrate | 67 | 70 | 41 | 35 | 26 | 20 |
| Fat | 4 | 10 | 24 | 45 | 35 | 60 |
| kcal/g | 3.9 | | 4.7 | | 5.2 | |
| **Ingredient** | **g** | **kcal** | **g** | **kcal** | **g** | **kcal** |
| Casein (80 mesh) | 200 | 800 | 200 | 800 | 200 | 800 |
| L-Cystine | 3 | 12 | 3 | 12 | 3 | 12 |
| DL-Methionine* | - | - | - | - | - | - |
| Corn Starch | 315 | 1260 | 73 | 291 | 0 | 0 |
| Maltodextrin 10 | 35 | 140 | 100 | 400 | 125 | 500 |
| Sucrose | 350 | 1400 | 173 | 691 | 69 | 275 |
| Cellulose (BW200) | 50 | 0 | 50 | 0 | 50 | 0 |
| Soybean Oil | 25 | 225 | 25 | 225 | 25 | 225 |
| Corn Oil* | - | - | - | - | - | - |
| Lard | 20 | 180 | 178 | 1598 | 245 | 2205 |
| Butter Fat* | - | - | - | - | - | - |
| Mineral Mix (S10001)* | - | - | - | - | - | - |
| Mineral Mix (S10026) | 10 | 0 | 10 | 0 | 10 | 0 |
| DiCalcium Phosphate | 13 | 0 | 13 | 0 | 13 | 0 |
| Calcium Carbonate | 6 | 0 | 6 | 0 | 6 | 0 |
| Sodium Chloride* | - | - | - | - | - | - |
| Potassium Citrate (1H_2_O) | 17 | 0 | 17 | 0 | 17 | 0 |
| Vitamin Mix (V10001) | 10 | 40 | 10 | 40 | 10 | 40 |
| Choline Bitartrate | 2 | 0 | 2 | 0 | 2 | 0 |
| FD&C Yellow Dye #5 | 0.05 | 0 | 0 | 0 | 0 | 0 |
| FD&C Red Dye #40 | 0 | 0 | 0.05 | 0 | 0 | 0 |
| FD&C Blue Dye #1 | 0 | 0 | 0 | 0 | 0.05 | 0 |
| **Total** | **1055** | **4057** | **858** | **4057** | **774** | **4057** |

* indicates ingredient in butter but not lard diets

10%, Research Diets D12450B

45%, Research Diets D12451

60%, Research Diets D12492

All values were obtained from Research Diets product data sheets and rounded to the nearest whole number where possible.

**Supplementary Table S2 │ Butter Diet Composition**

|  | **11%** | | **32%** | | **60%** | |
| --- | --- | --- | --- | --- | --- | --- |
|  | g% | kcal% | g% | kcal% | g% | kcal% |
| Protein | 16 | 17 | 19 | 17 | 22 | 17 |
| Carbohydrate | 71 | 73 | 57 | 51 | 31 | 23 |
| Fat | 5 | 11 | 16 | 32 | 35 | 60 |
| kcal/g | 3.9 | | 4.4 | | 5.3 | |
| **Ingredient** | **g** | **kcal** | **g** | **kcal** | **g** | **kcal** |
| Casein (80 mesh) | 190 | 760 | 190 | 760 | 190 | 760 |
| L-Cystine* | - | - | - | - | - | - |
| DL- Methionine | 3 | 12 | 3 | 12 | 3 | 12 |
| Corn Starch | 499 | 1994 | 215 | 860 | 0 | 0 |
| Maltodextrin 10 | 35 | 140 | 75 | 300 | 150 | 600 |
| Sucrose | 290 | 1160 | 290 | 1160 | 108 | 432 |
| Cellulose (BW200) | 30 | 0 | 30 | 0 | 30 | 0 |
| Soybean Oil* | - | - | - | - | - | - |
| Corn Oil | 39 | 354 | 118 | 1062 | 56 | 504 |
| Lard* | - | - | - | - | - | - |
| Butter Fat | 15 | 132 | 44 | 398 | 250 | 2250 |
| Mineral Mix (S10001) | 40 | 0 | 40 | 0 | 40 | 0 |
| Mineral Mix (S10026)* | - | - | - | - | - | - |
| DiCalcium Phosphate* | - | - | - | - | - | - |
| Calcium Carbonate | 6 | 0 | 6 | 0 | 6 | 0 |
| Sodium Chloride | 6 | 0 | 6 | 0 | 6 | 0 |
| Potassium Citrate (1H_2_O) | 14 | 0 | 14 | 0 | 14 | 0 |
| Vitamin Mix (V10001) | 11 | 44 | 11 | 44 | 11 | 44 |
| Choline Bitartrate | 2 | 0 | 2 | 0 | 2 | 0 |
| FD&C Yellow Dye #5 | 0.1 | 0 | 0 | 0 | 0.05 | 0 |
| FD&C Red Dye #40 | 0 | 0 | 0.1 | 0 | 0 | 0 |
| FD&C Blue Dye #1 | 0 | 0 | 0 | 0 | 0.05 | 0 |
| **Total** | **1178** | **4596** | **1043** | **4596** | **865** | **4602** |

* indicates ingredient in lard but not butter diets

11%, Research Diets D12489B

32%, Research Diets D12266B

60%, Research Diets D02101801

All values were obtained from Research Diets product data sheets and rounded to the nearest whole number where possible.
